# Decellularization of Human Pericardium with Potential Application in Regenerative Medicine

**DOI:** 10.5935/abc.20190130

**Published:** 2019-07

**Authors:** Estela de Oliveira Lima, Adriana Camargo Ferrasi, Andreas Kaasi

**Affiliations:** 1 Universidade Estadual Paulista Júlio de Mesquita Filho - FMB - Depto. Clínica Médica, Botucatu, SP - Brazil; 2 Santa Casa de Misericórdia de São Paulo - Instituto de Pesquisa, Inovação Tecnológica e Educação, São Paulo, SP - Brazil

**Keywords:** Regenerative Medicine, Pericardium, Tissue Scaffolds, Cells, Heart Valves/surgery, Translational Medical Research, Biocompatible Materials

Regenerative medicine is an interdisciplinary branch of the biomedical sciences that is showing, through the transposition of basic research into the clinic, great potential for therapeutic application, and the field is consequently becoming an important frontman for Translational Medicine. The essence of regenerative medicine consists of the repair or replacement of tissues and organs in which there is structural and functional deficiency. With a view of achieving this objective, several approaches have been proposed, including therapies that include genes, cells, biologic and synthetic *scaffolds*, which may or may not take part of a tissue engineering strategy.^1^ When dealing with synthetic scaffolds, it is possible to manipulate and control structural and mechanical properties, nonetheless, it is not possible to guarantee the same functional capacity as natural tissue.^2^ In addition, one of the great challenges is minimizing the risk of immunogenic reactions triggered by the repair scaffold’s composition.^3^ Owing to the challenges associated with the reestablishment on a functional and structural level of the cell microenvironment, the interest in scaffolds based on natural extracellular matrix (ECM) has increased considerably.

With a view of obtaining a biomaterial closely approximating tissues or organs having suffered damage, be it structural or functional, yet at the same time presenting safety from an immunological point of view, a new technology for clinical applications of ECM has been proposed - the decellularization of tissues and organs from human or animal donors. This technology makes use of physical, chemical and/or biochemical methods to eliminate cells from the target tissue/organ, be it xenogeneic or allogeneic, whose antigens represent an elevated risk of immunogenic reaction. The process seeks to ensure immunologic safety and the preservation of basic structural and functional components of the ECM, such as proteins, collagen and glycosaminoglycans (GAGs). The end product is a three-dimensional ECM scaffold with analogous shape to the original tissue, with preserved ultrastructural architecture and with greater biocompatibility and potential of tissue regeneration when compared to synthetic scaffolds.^4^

Pericardial tissue, mainly of bovine origin, has been widely used in cardiac surgery, whether for substitution of cardiac valves or for the repair of congenital disorders. Its fibroserous structure, rich in elastic fibres and collagen bundles, bestows upon the pericardium an elevated resistance to mechanical stress, and this resistance, together with the capability of uniform suture retention, represent essential characteristics for application in cardiovascular surgeries.^5,6^

With a view of reducing the probability of immunogenic response and rejection of the graft, the pericardium may be submitted to different treatments, among others the tissue fixation using glutaraldehyde, which is routinely used. While this method leads to lower immunogenicity of the tissue, the cytotoxicity and propensity of calcifications are two important inconveniences associated with this technique.^7,8^ Faced with these problems, other methods are being developed to improve on the safe utilization of pericardium in cardiac surgery, including decellularization. One of the most established techniques for this purpose is the treatment of the tissue using ionic detergents, such as sodium dodecyl sulfate (SDS). Whereas this decellularization agent is capable of removing undesirable natural components, such as antigenic molecules, it may also lead to structural alterations in the pericardial ECM; the loss of GAGs and important signaling proteins being notable examples. The structural damage caused by the treatment with SDS may impair the ideal *in situ* or *in vitro* recellularization of the scaffold, as well as altering the mechanical properties of the matrix, such as elasticity and extensibility.^9^

The original work presented by Wollmann et al.^10^ in this issue promotes the approach of human pericardium decellularization using a reduced quantity of detergent for application as a cardiovascular patch. The work presents itself with a view of minimizing the impact of the technique and to obtain a human ECM of better quality, non-toxic and that may retain as much as possible the original architecture and composition. The proposed methods exhibit a considerable removal of cell components, including DNA, the presence of which was reduced to approximately one-third of the original content. The tissue was also assessed with respect to cytotoxicity, and it was shown that no cytotoxic effects were seen *in vitro*. This observation brings a great stimulus for the even deeper investigation of the technique, such as the undertaking of *in vivo* tests with a view of future clinical application. Moreover, the treatment employed did not alter the composition of the pericardium with respect to the components elastin (qualitative assessment) and collagen (quantitative and qualitative assessment). The treatment did present a significant quantitative reduction in GAGs, which is well known from the literature when the decellularization is performed using SDS detergent (revised by Scarritt et al.^11^). Despite the structural changes with respect to GAGs, the biomechanical behavior of the matrix was similar to that of fresh tissue, and this enables use in the treatment of tissues that suffer intense mechanical loads, such as those encountered in the cardiovascular system.

We observe that one of the field’s great challenges is to unmask the best method of obtaining intact scaffolds. For that purpose, the concentration of the reagents can be modulated to guarantee the ideal ECM composition, and when it is optimized on the basis of the philosophy of “less is more”, the reagents fail considerably in removing completely cell debris, containing cellular DNA. Conversely, when the concentrations used are higher, the opposite is true.^12^. Considering that the success of the techniques of decellularization is directly associated to minimal change in composition, structure and mechanics of tissues, the work of Wollmann et al.^10^ is in line with the requirements of perfecting the methods of obtaining decellularized pericardium.

In the article, Wollmann et al.^10^ investigate mainly the effect of a concentration of SDS described as “low”, of 0.1% (w/v). That concentration is unquestionably low compared to the concentration of 1% used in, for example, decellularization of porcine kidneys.^13^ Other authors have adopted the descriptor “low concentration” for 0.5%, also for porcine kidneys.^14^ The concept of “low concentration” should be understood in the context of the tissue to be decellularized. For example, 0.1% may be a low concentration for a sub-millimetric tissue, such as pericardium, whereas 0.5% may equally be low for a centrimetric organ, such as the kidney. In addition to the challenge of customizing the concentration to the tissue to be decellularized, the variability between donors hampers the standardization of a scalable decellularization process and suggests that the path to clinical translation requires a customized and not necessarily standardized approach.

The use of patches in cardiac surgery is already a well-established practice, however, the most widely used biological material is of xenogeneic origin and is not submitted to the process of decellularization, increasing the risk of surgical reinterventions.^3,15^ The prospect of employing a decellularized pericardial patch, whose structural components are satisfactorily preserved and the antigenic molecules properly eliminated, is consequently of great interest for clinical application. These characteristics have the potential to enhance the signaling in the tissue for the recruitment and adhesion of cells from the recipient patient’s own tissue, to maintain ideal mechanical characteristics, in addition to reducing the immunogenicity of the graft.^16^ All this would elevate the durability of the graft, favor adequate tissue regeneration and reduce the propensity to fibrosis and calcification, improving the quality of life of the patient. Considering that cardiovascular disease is the driver of the main causes of death worldwide (31%),^17^ developing strategies that lead to products biocompatible for this application and more similar to natural tissue is of great importance to regenerative medicine and, as a consequence, new alternatives are emerging in the quest for an ideal cardiac biomaterial.
